# Information use and resource competition: an integrative framework

**DOI:** 10.1098/rspb.2015.2550

**Published:** 2016-02-24

**Authors:** Alexander E. G. Lee, James P. Ounsley, Tim Coulson, J. Marcus Rowcliffe, Guy Cowlishaw

**Affiliations:** 1 The Institute of Zoology, Zoological Society of London, Regent's Park, London, UK; 2 Department of Zoology, University of Oxford, Oxford, UK; 3 School of Biology, University of St Andrews, St Andrews, UK

**Keywords:** information use, social dominance, resource ecology, decision-making, producer–scrounger dynamics, individual differences

## Abstract

Organisms may reduce uncertainty regarding how best to exploit their environment by collecting information about resource distribution. We develop a model to demonstrate how competition can facilitate or constrain an individual's ability to use information when acquiring resources. As resource distribution underpins both selection on information use and the strength and nature of competition between individuals, we demonstrate interdependencies between the two that should be common in nature. Individuals in our model can search for resources either personally or by using social information. We explore selection on social information use across a comprehensive range of ecological conditions, generalizing the producer–scrounger framework to a wide diversity of taxa and resources. We show that resource ecology—defined by scarcity, depletion rate and monopolizability—determines patterns of individual differences in social information use. These differences suggest coevolutionary processes linking dominance systems and social information use, with implications for the evolutionary demography of populations.

## 1. Introduction

Organisms must secure resources such as food, mates and safety from predation to ensure survival and reproduction. Variability in the spatio-temporal distribution of these resources means that individuals face uncertainty regarding how best to exploit them. Individuals can thus acquire resources more efficiently by collecting uncertainty-reducing information [1,2]. However, resource distribution also underpins the strength and nature of competition between individuals [3–5]. Despite this convergence in resource distribution as a selective force, very little is known about how competition might facilitate or constrain information use. In this study, we present a general model to predict how resource competition will affect individual information use across a comprehensive range of ecological conditions, generating novel insights into the behavioural processes underlying the evolution of social systems.

Information can be gathered either personally, by direct interaction with the environment, or socially, by observing the behaviours of others [6,7]. The majority of research into information use has focused on two key ways that the costs and benefits of ‘personal’ versus ‘social’ information can differ. First, empirical and theoretical studies have shown that if gathering personal information involves long search times, trial-and-error sampling or risk-taking, collecting social information can reduce these costs by exploiting the efforts of others [8–10]. Second, the usefulness of one type of information over the other will depend on how rapidly each becomes outdated [11–13]. The resultant cost/reliability trade-off between personal and social information suggests that selection should favour strategies that balance adaptively an individual's reliance on each source [7,14].

Individuals may nonetheless be constrained in their ability to use information due to competition with others over resources, but such limitations have received little attention. An individual may be free to *collect* reliable social information at a relatively low cost, yet its ability to *use* it may be mediated by certain phenotypic factors, such as competitive ability. For example, if food patches or breeding territories are both limited and monopolizable, then the advantages of using social information may be restricted to socially dominant individuals (e.g. [15–17]). Since social information has been variously implicated as a regulator of population growth [18], a benefit of group living [19] and a facilitator of learning and culture [20,21], constraints on its use may have important ecological and evolutionary consequences.

The producer–scrounger model represents a useful game theoretical framework for exploring the influence that competition over resources can have on the evolutionary dynamics of social information use [9,22]. The model considers groups of individuals searching for and consuming discrete resource patches by using either personal information (‘producing’) or social information (‘scrounging’). When playing ‘producer’, an individual searches for its own resources; when playing ‘scrounger’, it looks for and exploits the discoveries of producers [23]. A well-established insight from producer–scrounger games, relating to one element of resource distribution, is that the ‘finder's share’ should influence optimal levels of social information use within a group [24]. When producers can fully deplete a discovery before the arrival of any scroungers—at which point social information becomes outdated—there is no benefit to scrounging behaviour. By contrast, when depletion time is longer, the finder's share is lower and scrounging becomes more prevalent. Consistent with this, Giraldeau & Livoreil [25] experimentally demonstrated a positive relationship between the finder's share and scrounging behaviour in nutmeg mannikins (*Lonchura punctulata*).

Two further elements of resource distribution can readily be incorporated into producer–scrounger games, but have received limited attention. First, almost all producer–scrounger models assume that resources are so hard to find that they are only discovered singly and successively, regardless of the number of individuals searching for them as producers. However, it is clear that many organisms inhabit environments in which simultaneous resource discoveries are likely. Those few studies that have varied the difficulty of resource discovery such that simultaneous resource discoveries can occur [26–28] propose that social information use should decrease as finding resources using personal information becomes easier. Second, only one model [29] has explored the effects of resource monopolizability on scrounging behaviour, while others have assumed scramble-like competition between a producer and any scroungers at its discovery. Barta & Giraldeau [29] demonstrated a positive link between dominance and scrounging behaviour when resources were monopolizable.

No study to date has attempted to combine the three elements of resource distribution outlined above to better understand how their influences on the strength and nature of competition might interact to impact individual information use. Such an approach should be of general interest given the range of ecological conditions that different taxa experience when exploiting resources in nature. Here, we expand the producer–scrounger game to develop a general model for how resource ecology should affect individual information use in a social context. We define resource ecology along three axes of variation: (i) the ‘scarcity’ of resource patches, which quantifies how difficult they are to discover using personal information; (ii) the ‘depletion rate’ of resource patches, which quantifies how rapidly social information becomes outdated following discovery; and (iii) the ‘monopolizability’ of resource patches, which quantifies the degree to which competitors can exclude one another based on differences in competitive ability [30]. Within this framework, the classic producer–scrounger game is represented by a specific subset of the environmental conditions considered.

Using this framework, we investigate how individual decisions to use personal or social information may be affected or constrained by ecological conditions and individual phenotype. By considering the interactive effects of three dimensions of resource ecology, we provide general insights into how the relative costs of not just collecting, but also using, personal versus social information should influence individual information use. Furthermore, we demonstrate that the individual benefits of high competitive ability are dependent on more than just resource monopolizability when individuals face uncertainty about the spatio-temporal distribution of resources. As such, patterns of resource ecology may have previously unexpected influences on the character of social systems across taxa.

## 2. The model

We model groups of *N* individuals searching for and consuming resource patches. Patches contain *F* resource units. Individuals can choose to search for patches using one of two mutually exclusive tactics: producing or scrounging. Producers collect and use personal information, sampling their environment asocially. The per capita resource discovery rate for producers in a given ‘time step’ is determined by *λ*, which ranges from 0 to 1 and represents ‘scarcity’. The lower the value of *λ*, the more difficult resources are to find. Scroungers, in contrast, collect and use social information. They do not contribute to the group's resource discovery rate, but exploit patches produced by others. The proportion of the group producing and scrounging is denoted by *q* and (1 − *q*), respectively.

The total number of patches discovered per time step is given by *λqN*—the per capita resource discovery rate for producers multiplied by the number of producers in the group. Simultaneous resource discoveries occur if *λqN* > 1, but individual scroungers can only access a maximum of one discovered patch per time step. Therefore, when *λqN* ≥ 1, *λ* represents the ratio of the costs of collecting social versus personal information, since a scrounger can access a patch each time step, whereas a producer discovers a patch only every 1/*λ* time steps. When *λqN* < 1, however, scroungers only access a patch every 1/(*λqN*) time steps. In this case, the ratio of the costs of collecting social versus personal information will be 1/*qN*, since scroungers access each producer's discovery, while producers only benefit from their own discoveries. Because our parameters and variables do not change across time steps for a given group composition, all resource consumption formulae given below represent rates per time step (i.e. *T* = 1).

A producer discovering a patch gains a finder's advantage of *a* resource units before any scroungers arrive [24]. The remaining (*F* − *a*) resource units, defined as *A*, are divided between the producer and any scroungers present in proportion to their relative competitive weights. Patches are fully depleted in the same time step that they are discovered, meaning that the finder's share (*a*/*F*) represents the ‘depletion rate’ of a resource patch within that time step. An individual's competitive weight is defined as2.1
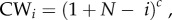
where *i* denotes an individual's ranked competitive ability relative to others in the group (expressed as an integer, ascending from 1 to *N*; hereafter ‘social rank’), and *c* defines the degree to which patches can be monopolized [29].

The exponent *c* represents resource ‘monopolizability’, and may be best considered as patch area. When *c* = 0, an individual cannot monopolize a patch regardless of social rank; CW*_i_* is the same for all individuals, and competition at patches is scramble-like. However, as *c* increases, resources become more defensible and the degree to which social rank influences competitive asymmetry between individuals increases following a power law. When *c* = 100, the highest-ranking individual in a patch essentially monopolizes *A*.

If *λqN* ≤ 1, scroungers are able to access each patch discovery. In this case, the rate of resource consumption for producers and scroungers is equivalent to that given by Barta & Giraldeau [29]. When, in addition to this, *c* = 0, the model becomes equivalent to the classical producer–scrounger game [23,24]. The rate of resource consumption for the *i*th-ranking producer with competitive weight CW*_i_* when *λqN* ≤ 1 is given by2.2
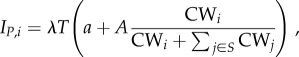
where *S* is the set of social ranks for all (1 − *q*)*N* individuals playing scrounger, and CW*_j_* is the competitive weight of the *j*th scrounger in the set, making 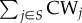 the summed competitive weight of all scroungers in the group. The rate of resource consumption for the *i*th-ranking scrounger, in contrast, is2.3
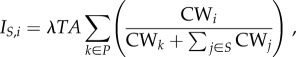
where *P* is the set of social ranks for all *qN* individuals playing producer, making CW*_k_* the competitive weight of the *k*th producer in the set. The summation operator describes a scrounger accessing the discoveries of all *qN* producers in the group.

When *λqN* > 1, scroungers cannot access all discoveries, since some discoveries are occurring simultaneously. As such, we assume that the probability of a scrounger exploiting a given producer's discovery in each time step is determined by the total number of discoveries in the group, which we take to be the expected value of *λqN*. While *λqN* will in reality also be probabilistic, modelling its expected (i.e. mean) value will not have affected the model's overall predictions, but allowed us to focus on how scroungers may be distributed probabilistically across different producers. A given producer is at risk of being exploited by a total of *n* = (1 − *q*)*N* mutually independent scroungers, where the likelihood of each of these scroungers being present is thus 1/*λqN*. The probability of a given producer being scrounged by a certain number, *l*, of the *n* scroungers is drawn from a binomial distribution. However, the identities of the *l* scroungers exploiting a producer will influence the cumulative competitive weight of the individuals within a patch. We thus calculate the probability of occurrence and cumulative competitive weight for all 2*^n^* possible combinations of scroungers that can occur at a given producer's discovery. We define a 2*^n^* × *n* matrix, **S**_P_, which defines the absence (0) or the presence (1) of each scrounger for each combination. The probability of occurrence for each of these combinations is given in a 2*^n^* × 1 column vector, **B**_P_, the elements of which are calculated as2.4
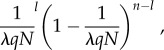
where the value of *l* for each row is the sum of the elements in the corresponding row in **S**_P_. To calculate the cumulative competitive weight of scroungers for each combination, we multiplied **S**_P_ by a *n* × 1 column vector, **C**_P_, of competitive weights for each individual scrounger, to produce a 2*^n^* × 1 column vector, **W**_P_, of competitive weights summed across scroungers for each possible combination of individual scroungers present.

As such, when *λqN* > 1, the rate of resource consumption for the *i*th-ranking producer is given by2.5
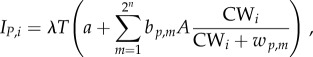
where *b_p,m_* and *w_p,m_* represent the *m*th elements (i.e. rows) of the **B**_P_ and **W**_P_, vectors, respectively, and *m* ranges from 1 to 2*^n^*.

To calculate the rate of resource consumption for a given scrounger, we must define all of the possible contexts in which it can be when exploiting producers. For each context, we calculate its probability of occurrence and the cumulative competitive weight of individuals present. A scrounger will always exploit one producer when *λqN* > 1. This may occur alone or in conjunction with a certain number, (*l* − 1), of the remaining (*n* − 1) scroungers. The total number of possible combinations of other scroungers occurring alongside a given focal scrounger is 2^(*n*−1)^. We define a 2^(*n*−1)^ × (*n* − 1) matrix, **S**_S_, which records the absence (0) or the presence (1) of the remaining scroungers for each combination. The likelihood of any individual scrounger exploiting a given producer is 1/*λqN*, making the probability of a given scrounger sharing a resource with a given combination of the remaining scroungers2.6
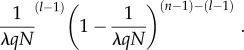


Since the focal scrounger will itself only have a 1/*λqN* chance of being present at a given producer's discovery, the probability of each of these combinations occurring at a given discovery will be2.7
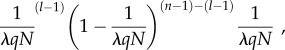
simplified as2.8
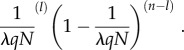


We calculate this probability for each of the 2^(*n*−1)^ possible combinations to produce a 2^(*n*−1)^ × 1 column vector, **B**_S_.

The identity of a given focal scrounger will influence the cumulative competitive weight of the (*n* − 1) remaining scroungers. As such, we define, for each focal scrounger, a separate (*n* − 1) × 1 column vector, **C**_S*i*_, of competitive weights for the remaining scroungers, where *i* is the rank of the focal scrounger. The cumulative competitive weight of remaining scroungers for each of the 2^(*n*−1)^ combinations is calculated for the *i*th-ranking scrounger by multiplying the matrix **S**_S_ with the column vector **C**_S*i*_, to generate a 2^(*n*−1)^ × 1 column vector, **W**_S*i*_.

When *λqN* > 1, therefore, the rate of resource consumption for the *i*th-ranking scrounger is given by2.9
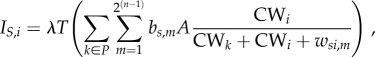
where *b_s,m_* and *w_si,m_* represent the *m*th elements (i.e. rows) of the **B**_S_ and **W**_S*i*_ vectors, respectively, and *m* ranges from 1 to 2^(*n*−1)^. The competitive weight of a given producer, CW*_k_*, is defined as above.

We analysed the model—for fixed values of *N*, *a*/*F*, *λ* and *c*—by allowing individuals to switch between the producer and scrounger tactic in an attempt to improve their relative rate of resource consumption, or relative fitness. This was calculated for the *i*th-ranking individual as2.10
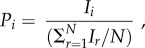
its fitness relative to the group's average. In this way, we searched for Nash equilibria: stable group compositions where no individual could increase its payoff by switching tactic. We generated results numerically across a range of group sizes (4, 8, 16), but our equations relate to groups of any size. Here, we present only those findings where *N* = 16, since the other group sizes modelled always showed qualitatively similar patterns. The robustness of our findings to increasing group size is corroborated by our analysis of a larger range of group sizes (ranging from 4 to 28) across a pertinent range of the parameter space, the results of which are presented in the electronic supplementary material.

We began each model run by defining a group as a vector of zeros and ones of length *N*, where the value at position *i* represents the tactic (0 = producer; 1 = scrounger) of the *i*th-ranking individual [29]. Initial group composition was generated such that each individual had a 50% chance of starting as a producer or scrounger. The group was then perturbed by randomly selecting one individual to switch tactics. If this individual's payoff increased, the new tactic would be maintained; otherwise, it would revert to its previous tactic. This perturbation was repeated until a Nash equilibrium was reached. Model runs for a given parameter set were replicated up to 10 000 times in order to account for multiple Nash equilibria. We thus calculated—for each group and each individual in each parameter set—the probability of scrounging based on average tactics in the stable group compositions of model replicates. All model analysis was conducted in R v. 3.0.2 [31].

## 3. Results

**Figure 1.**
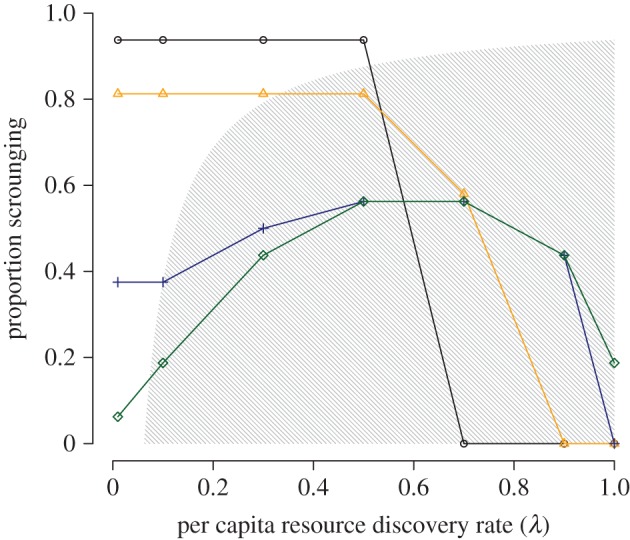
The combined influence of resource scarcity and monopolizability on levels of scrounging in a group. Simultaneous resource discoveries occurred only within the shaded region (i.e. where *λqN* > 1). Values of *c* as follows: 0 (black circles), 1 (orange triangles), 10 (blue crosses), 100 (green diamonds). *N* = 16; *a*/*F* = 0.05.

**Figure 2.**
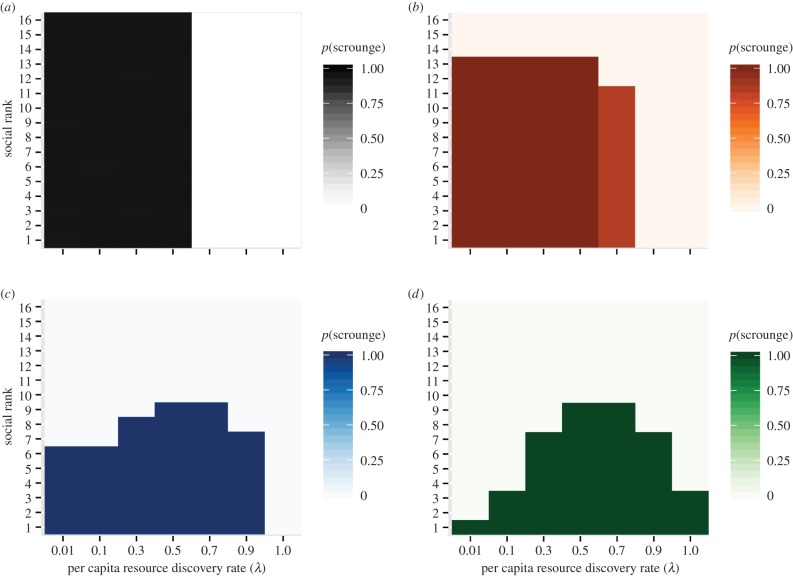
The combined effect of resource scarcity and monopolizability on the probability of scrounging for individuals of different social rank. Resource monopolizability for each panel as follows: (*a*) *c* = 0 (black), (*b*) *c* = 1 (orange), (*c*) *c* = 10 (blue) and (*d*) *c* = 100 (green). Colour coding corresponds to that given in figure 1. Note that social rank ranges highest to lowest from 1 to *N*. *N* = 16; *a*/*F* = 0.05.

Increasing the producer's resource discovery rate (*λ*) reduced the relative cost of collecting personal information. However, the impact of this relationship on the decision to produce or scrounge was dependent upon resource monopolizability. When individuals were unable to monopolize resources (i.e. *c* = 0), scrounging became less common if patches were easier to find (higher *λ*) (figure 1). Under these conditions, there was no between-individual variation in scrounging behaviour (figure 2*a*). Scrounging ultimately disappeared from the population (i.e. producing became fixed) when resources were sufficiently easy to find using personal information that the associated costs of resource sharing were no longer economical.

As resources became monopolizable (increasing *c*), the relationship between the discovery rate and the proportion of scrounging in a population changed dramatically. First, when the discovery rate was low, such that no more than one patch was being discovered per time step (*λqN* ≤ 1), scrounging became less common at higher values of *c* (figure 1). This occurred because the more effectively every discovery could be monopolized by a single, top-ranked individual playing scrounger, the less profitable this tactic became for others (figure 2*a–d*, far left columns). Rather, other individuals would benefit more from playing producer: even though patch discoveries would be rare, and would be largely appropriated by the dominant scrounger, producers would at least secure a finder's advantage.

Second, under conditions of strong monopolizability (*c* ≥ 10), intermediate discovery rates promoted higher levels of scrounging despite the lower relative cost of collecting personal information (figure 1). This was because simultaneous resource discoveries (i.e. *λqN* > 1) could occur more easily as *λ* increased. Simultaneous resource discoveries precluded complete monopolization by single individuals, freeing up resources for other individuals to scrounge. Owing to the higher competitive weights of higher-ranking individuals, however, scrounging remained tightly linked to social rank despite more individuals being able to use the tactic (figure 2*c,d*). Finally, scrounging began to decline once resources became so easy to find that even the highest-ranked individuals would benefit more from producing—thus gaining a finder's advantage—than from monopolizing the discoveries of others (figure 1). The overall result when *c* ≥ 10 was thus a ‘peaked’ relationship between resource discovery rate and population levels of scrounging.

**Figure 3.**
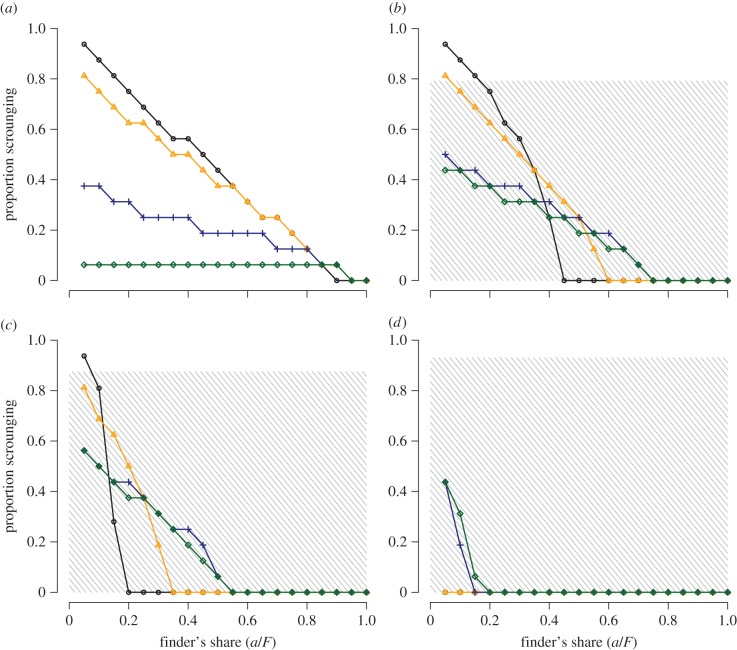
The effect of the finder's share on levels of scrounging in a group depends on interactions between resource scarcity and monopolizability. Panels show data for different values of per capita resource discovery rate (*λ*) as follows: (*a*) 0.01, (*b*) 0.3, (*c*) 0.5 and (*d*) 0.9. Values of *c* as follows: 0 (black circles), 1 (orange triangles), 10 (blue crosses), 100 (green diamonds). Simultaneous resource discoveries occurred only within the shaded regions (i.e. where *λqN* > 1). *N* = 16; *a*/*F* = 0.05.

Scrounging was less common when the finder's share was high (figure 3). Individuals were less likely to scrounge as those producing benefited from consuming greater portions of their discoveries, leaving less for scroungers to exploit. The strength of this effect, however, was influenced by both resource discovery rate and monopolizability. While at very small finder's shares the levels of scrounging were usually lower when monopolizability was high, the rate of decline of scrounging with increasing finder's share was actually slower at higher levels of monopolizability (figure 3*a–d*). This reflects the fact that scroungers able to monopolize patches were more robust to the losses associated with a higher finder's share, because they still secured large proportions of the remaining *A* resource units. By contrast, the general effect of a higher resource discovery rate was to accelerate the decline in scrounging associated with the finder's share (figure 3*a–d*). When resources could be discovered more easily, individuals benefited from producing when the finder's share was high.

Finally, high resource monopolizability allowed scrounging to persist at intermediate values of the finder's share when it would otherwise disappear from the population (figure 3*b*), although this effect persisted only at progressively smaller values of the finder's share as discovery rates increased (figure 3*c,d*). This was caused by a combination of simultaneous resource discoveries and the robustness of scrounging for individuals of high social rank described above.

**Figure 4.**
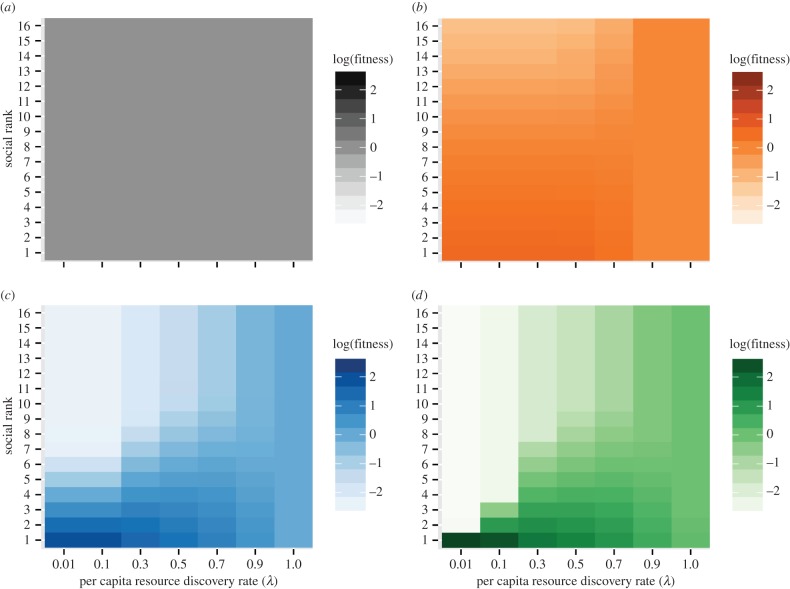
Resource scarcity and monopolizability interact to determine both the skew and strength of the fitness benefits of social rank. Resource monopolizability for each panel as follows: (*a*) *c* = 0 (black), (*b*) *c* = 1 (orange), (*c*) *c* = 10 (blue) and (*d*) *c* = 100 (green). Colour coding corresponds to that given in figure 1. Note that social rank ranges highest to lowest from 1 to *N*. *N* = 16; *a*/*F* = 0.05.

The interactive effects of resource discovery rate and monopolizability on scrounging behaviour had important fitness consequences (figure 4). The exclusivity of scrounging to individuals of high rank when resources were monopolizable but extremely rare resulted in a strong skew in fitness favouring dominant individuals. This increased in proportion with *c*, and tended towards a single, top-ranked scrounger with a very high relative fitness (figure 4*d*, far-left column). As resources became easier to find, however, this fitness skew became less dramatic (figure 4*d*). This was primarily due to the occurrence of simultaneous resource discoveries, which made scrounging behaviour and its benefits more evenly shared across more individuals of relatively high social rank, and lower-ranked producers less prone to being in direct competition with the highest ranked individuals. Low-ranked producers also benefited from more frequent discoveries, and the resulting finder's share benefits, when the discovery rate was high. These findings were in stark contrast to the outcome when resources could not be monopolized (*c* = 0). In these cases, there was no variation in scrounging propensity between individuals, leading the population to a stable mix of producers and scroungers where all individuals had equal fitness irrespective of social rank or resource scarcity (figure 4*a*).

**Figure 5.**
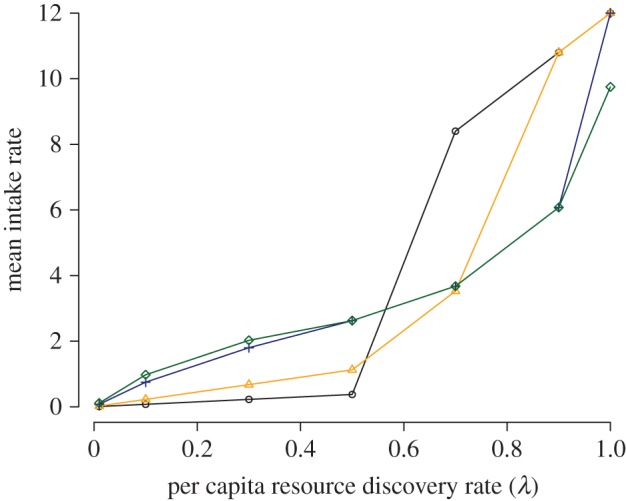
Resource scarcity and monopolizability affects a group's average resource consumption rate through their influence on scrounging behaviour. Values of *c* as follows: 0 (black circles), 1 (orange triangles), 10 (blue crosses), 100 (green diamonds). *N* = 16; *a*/*F* = 0.05.

Since scroungers do not contribute to resource discovery, they can reduce a population's average individual resource consumption rate, potentially impacting on various demographic processes. When resources were rare (i.e. low *λ*), higher values of *c* resulted in fewer scroungers and therefore greater average intake rates (figure 5). However, as resources became more common this pattern reversed: high values of *c* led to more scroungers and thus lower average intake rates relative to when *c* was low.

## 4. Discussion

Our aim was to explore the interdependencies between information use and competition over resources, driven by resource distribution as a shared selective force. To do this, we investigated how three key aspects of resource ecology—scarcity, depletion rate and monopolizability—interact to promote or constrain the use of social information when acquiring resources in an uncertain environment. Further, we asked how such influences might lead to variation in fitness between individuals through differential access to resources. Our findings generated two key predictions. First, the effects of resource scarcity on social information use should depend strongly on resource monopolizability. Second, the potential benefits of social dominance should be closely linked to social information use in uncertain environments, determined not just by monopolizability, but all three aspects of resource ecology. Below, we discuss these predictions in the context of previous work and propose avenues for future research. We then raise an important theoretical consideration for the study of social information use, and conclude by outlining the potential evolutionary demographic implications of our research.

Previous producer–scrounger models have proposed that individuals should rely more on social information when the costs of collecting personal information are high (i.e. scrounging increases as valuable resources become harder to find) [26–28,32]. Although this relationship seems intuitive, direct experimental support is surprisingly lacking [14], possibly because the true relationship is dependent on the degree of resource monopolizability. Thus, our results confirmed this pattern when resources were not monopolizable, but predicted a ‘peaked’ relationship when resources were monopolizable, where social information use (scrounging) only increases initially but then declines again as resources become progressively scarcer. Data from Koops & Giraldeau [33] provides circumstantial evidence supporting this explanation. These authors found that scrounging declined in European starlings (*Sturnus vulgaris*) when novel food patches were more scarce, contrary to conventional wisdom but consistent with the downturn our model predicts at higher levels of resource scarcity and monopolizability. Crucially, starling social groups exhibit a dominance structure [34], and Koops & Giraldeau [33] reported that scroungers in their study were primarily socially dominant starlings with a competitive advantage at resource patches. Our findings thus suggest that resource scarcity, depletion rate and monopolizability should be considered in unison when making predictions about how selection should act on social information use in a given species.

Most research into information use has focused on how individuals optimize their reliance on social versus personal information based on trade-offs between their collection costs and reliabilities [14,20], and their negatively frequency-dependent payoffs [23]. Very little attention has been given to the constraints that competition imposes on an individual's liberty to use social information to access resources. Our findings are consistent with the only previous study to consider systematically how variation in competitive ability might constrain individual social information use due to competition over limited resources [29]: when resources are monopolizable, social information use may become exclusive to dominant individuals, leading to a positive relationship between social rank and resource acquisition rate. However, our model further demonstrates that resource scarcity is critical in determining the degree of this exclusivity. Specifically, it is only under conditions of sequential resource discovery that a single individual can make exclusive use of social information; when resources are discovered simultaneously social information may be exploited by successively lower ranked individuals.

Despite the clear influence that social dominance may play in constraining information use, we know of no studies exploring how social information use varies in response to systematic manipulation of resource monopolizability in taxa exhibiting dominance hierarchies. Liker & Barta [35] showed that dominant house sparrows (*Passer domesticus*) scrounged more than subordinates when searching for spatially clumped seeds, but did not investigate conditions where resources could not be monopolized. A number of other experimental producer–scrounger studies in birds and primates have reported either a positive relationship or no relationship between social rank and scrounging [17,36,37]. Consistent with our findings, observational studies in chacma baboons have shown that dominance-linked scrounging increases when food patches are monopolizable [16,38]. Our model suggests that predictions (and associated experimental designs) regarding the relationship between social dominance and information use for a given taxon should be guided by an appreciation of resource scarcity, depletion rate and monopolizability. Further empirical work is needed to experimentally test the predictions that resource monopolizability constrains an individual's use of social information according to dominance, leading to differential access to resources, and that the patterns of these constraints is dependent on resource scarcity.

The fact that resource monopolizability influences the benefits of dominance is well established [30,39,40]. However, since there is expected to be uncertainty associated with the spatio-temporal distribution of most resources, our model highlights a crucial role for social information use in capturing the benefits of dominance. As such, selection pressures on dominance will depend on multidimensional aspects of resource ecology (e.g. scarcity, depletion rate and monopolizability) that influence both the benefits of social information use and competition between individuals. This does not mean that we predict the ecology of any single resource to lead to any particular information use phenotype or social system, since organisms must exploit many different resources to survive and reproduce. Overall selection on these phenotypes and systems will be driven by the combined pressures of multiple resources ecologies through space and time [41].

Our findings also highlight an important theoretical issue that requires development if we are to fully understand the evolutionary ecology of social information use. We only consider the acquisition of ephemeral resources that are fully depleted upon discovery. Like most previous theory, we thus assume that any information generated by the discovery of a resource becomes useless upon its depletion. Yet, it is clear that many organisms exploit resources that exhibit at least some spatio-temporal predictability, such that previous experience, or prior information, can be used to inform future decisions [10,42–44]. When information is reusable or generalizable in this way, the relationship between social information use and competition over resources may change dramatically. Specifically, social information use may be decoupled from the context in which it was collected in a way that would not be possible with unpredictable, ephemeral resources (where information must be used as soon as it is collected). For example, an individual may be able to collect social information in a highly competitive situation and then use it in a less competitive one. In this way, competitive constraints on social information use may be relaxed or altered. Since the fitness benefits of information use are generally expected to be associated with improvements in resource exploitation, research is needed to more formally define the links between information acquisition and resource acquisition, and to explore how these links can vary.

Our model may have important evolutionary demographic implications. Coolen *et al.* [18] showed that social information use can regulate population dynamics. They argued that, because individuals forgoing personal information in favour of social information (i.e. scroungers) do not contribute to per capita food discovery rates, higher levels of scrounging could reduce average population birth rates [18]. Our results thus suggest that demographic rates may differ for different systems of social dominance—ranging from egalitarian to despotic—driven by differences in levels of scrounging behaviour within groups, in turn driven by differences in resource ecology. For example, when simultaneous resource discoveries occur, we predict scrounging to be more prevalent if resources are monopolizable, resulting in lower average resource consumption rates. We predict the opposite pattern when resources are discovered sequentially. It is widely accepted that population-level processes such as density dependence and trait-mediated interference are often driven by underlying behavioural mechanisms [45–47]. Better understanding of the relationships between resource ecology, information use, social dominance and fitness should thus strengthen our understanding of the differences in population dynamics across environments and taxa, and improve our ability to predict population responses to environmental change.

## Supplementary Material

Electronic Supplementary Material

## Data accessibility

Data are available to download from Dryad: http://dx.doi.org/10.5061/dryad.mn36c.

## References

[1] Dall SRX, Giraldeau L-A, Olsson O, McNamara JM, Stephens DW 2005 Information and its use by animals in evolutionary ecology. Trends Ecol. Evol. 20, 187–193. (10.1016/j.tree.2005.01.010) 16701367

[2] McNamara JM, Dall SRX 2010 Information is a fitness enhancing resource. Oikos 119, 231–236. (10.1111/j.1600-0706.2009.17509.x)

[3] Parker GA 2000 Scramble in behaviour and ecology. Phil. Trans. R. Soc. B 355, 1637–1645. (10.1098/rstb.2000.0726) 11127910 PMC1692901

[4] Milinski M, Parker GA 1991 Competition for resources. In Behavioural ecology: an evolutionary approach (eds Krebs J, Davies N), pp. 137–168. Oxford, UK: Blackwell Scientific Publications.

[5] Stillman RA, Caldow RWG, Goss-Custard JD, Alexander MJ 2000 Individual variation in intake rate: the relative importance of foraging efficiency and dominance. J. Anim. Ecol. 69, 484–493. (10.1046/j.1365-2656.2000.00410.x)

[6] Danchin E, Giraldeau L-A, Valone TJ, Wagner RH 2004 Public information: from nosy neighbors to cultural evolution. Science 305, 487–491. (10.1126/science.1098254) 15273386

[7] Rieucau G, Giraldeau L-A 2011 Exploring the costs and benefits of social information use: an appraisal of current experimental evidence. Phil. Trans. R. Soc. B 366, 949–957. (10.1098/rstb.2010.0325) 21357217 PMC3049093

[8] Rendell L et al. 2010 Why copy others? Insights from the social learning strategies tournament. Science 328, 208–213. (10.1126/science.1184719) 20378813 PMC2989663

[9] Giraldeau L-A, Dubois F 2008 Social Foraging and the Study of Exploitative Behavior. Adv. Study Behav. 38, 59–104. (10.1016/S0065-3454(08)00002-8)

[10] Kendal RL, Coolen I, Laland KN 2004 The role of conformity in foraging when personal and social information conflict. Behav. Ecol. 15, 269–277. (10.1093/beheco/arh008)

[11] Boyd R, Richerson PJ 1985 Culture and the evolutionary process. Chicago, IL: University of Chicago Press.

[12] Kameda T, Nakanishi D 2002 Cost–benefit analysis of social/cultural learning in a nonstationary uncertain environment. Evol. Hum. Behav. 23, 373–393. (10.1016/S1090-5138(02)00101-0)

[13] van Bergen Y, Coolen I, Laland KN 2004 Nine-spined sticklebacks exploit the most reliable source when public and private information conflict. Proc. R. Soc. Lond. B 271, 957–962. (10.1098/rspb.2004.2684) PMC1691685 15255051

[14] Kendal RL, Coolen I, Laland KN 2009 Adaptive trade-offs in the use of social and personal information. In Cognitive ecology II (eds Dukas R, Ratcliffe JM), pp. 249–271. Chicago, IL: University of Chicago Press.

[15] Piper WH, Tischler KB, Klich M 2000 Territory acquisition in loons: the importance of take-over. Anim. Behav. 59, 385–394. (10.1006/anbe.1999.1295) 10675261

[16] King AJ, Isaac NJB, Cowlishaw G 2009 Ecological, social, and reproductive factors shape producer-scrounger dynamics in baboons. Behav. Ecol. 20, 1039–1049. (10.1093/beheco/arp095)

[17] Stahl J, Tolsma PH, Loonen MJJE, Drent RH 2001 Subordinates explore but dominants profit: resource competition in high Arctic barnacle goose flocks. Anim. Behav. 61, 257–264. (10.1006/anbe.2000.1564) 11170715

[18] Coolen I, Giraldeau LA, Vickery W 2007 Scrounging behavior regulates population dynamics. Oikos 116, 533–539. (10.1111/j.2006.0030-1299.15213.x)

[19] Clark CW, Mangel M 1984 Foraging and flocking strategies: information in an uncertain environment. Am. Nat. 123, 626–641. (10.1086/284228)

[20] Laland KN 2004 Social learning strategies. Learn. Behav. 32, 4–14. (10.3758/BF03196002) 15161136

[21] Whiten A, Hinde RA, Laland KN, Stringer CB 2011 Culture evolves. Phil. Trans. R. Soc. B 366, 938–948. (10.1098/rstb.2010.0372) 21357216 PMC3049105

[22] Barnard CJ 1984 Producers and scroungers: strategies of exploitation and parasitism. London, UK: Chapman & Hall.

[23] Vickery WL, Giraldeau L-A, Templeton JJ, Kramer DL, Chapman CA 1991 Producers, scroungers, and group foraging. Am. Nat. 137, 847–863. (10.1006/anbe.1996.0014)

[24] Barnard CJ, Sibly RM 1981 Producers and scroungers: a general model and its application to captive flocks of house sparrows. Anim. Behav. 29, 543–550. (10.1016/S0003-3472(81)80117-0)

[25] Giraldeau L-A, Livoreil B 1998 Game theory and social foraging. In Game theory and animal behavior (eds Dugatkin LA, Reeve HK), pp. 16–37. New York, NY: Oxford University Press.

[26] Ohtsuka Y, Toquenaga Y 2009 The patch distributed producer-scrounger game. J. Theor. Biol. 260, 261–266. (10.1016/j.jtbi.2009.06.002) 19501599

[27] Beauchamp G, Giraldeau L-A 1996 Group foraging revisited: information sharing or producer–scrounger game? Am. Nat. 148, 738–743. (10.1086/285951)

[28] Afshar M, Giraldeau L-A 2014 A unified modelling approach for producer–scrounger games in complex ecological conditions. Anim. Behav. 96, 167–176. (10.1016/j.anbehav.2014.07.022)

[29] Barta Z, Giraldeau L-A 1998 The effect of dominance hierarchy on the use of alternative foraging tactics: a phenotype-limited producing–scrounging game. Behav. Ecol. Sociobiol. 42, 217–223. (10.1007/s002650050433)

[30] Parker GA 1974 Assessment strategy and the evolution of fighting behaviour. J. Theor. Biol. 47, 223–243. (10.1016/0022-5193(74)90111-8) 4477626

[31] R Core Team. 2013 R: a language and environment for statistical computing. Vienna, Austria: R Foundation for Statistical Computing.

[32] Beauchamp G 2008 A spatial model of producing and scrounging. Anim. Behav. 76, 1935–1942. (10.1016/j.anbehav.2008.08.017)

[33] Koops MA, Giraldeau L-A 1996 Producer–scrounger foraging games in starlings: a test of rate-maximizing and risk-sensitive models. Anim. Behav. 51, 773–783. (10.1006/anbe.1996.0082)

[34] Summers RW, Westlake GE, Feare CJ 1986 Differences in the ages, sexes and physical condition of starlings *Sturnus vulgaris* at the centre and periphery of roosts. Ibis 129, 96–102. (10.1111/j.1474-919X.1987.tb03164.x)

[35] Liker A, Barta Z 2002 The effects of dominance on social foraging tactic use in house sparrows. Behaviour 139, 1061–1076. (10.1163/15685390260337903)

[36] Beauchamp G 2006 Phenotypic correlates of scrounging behavior in zebra finches: role of foraging efficiency and dominance. Ethology 112, 873–878. (10.1111/j.1439-0310.2006.01241.x)

[37] di Bitetti MS, Janson CH 2001 Social foraging and the finder's share in capuchin monkeys, *Cebus apella*. Anim. Behav. 62, 47–56. (10.1006/anbe.2000.1730)

[38] Marshall HH, Carter AJ, Coulson T, Rowcliffe JM, Cowlishaw G 2012 Exploring foraging decisions in a social primate using discrete-choice models. Am. Nat. 180, 481–495. (10.1086/667587) 22976011

[39] Vehrencamp SL 1983 A model for the evolution of despotic versus egalitarian societies. Anim. Behav. 31, 667–682. (10.1016/S0003-3472(83)80222-X)

[40] Vahl WK, Lok T, van der Meer J, Piersma T, Weissing FJ 2005 Spatial clumping of food and social dominance affect interference competition among ruddy turnstones. Behav. Ecol. 16, 834–844. (10.1093/beheco/ari067)

[41] Botero CA, Weissing FJ, Wright J, Rubenstein DR 2015 Evolutionary tipping points in the capacity to adapt to environmental change. Proc. Natl Acad. Sci. USA 112, 184–189. (10.1073/pnas.1408589111) 25422451 PMC4291647

[42] Marshall HH, Carter AJ, Ashford A, Rowcliffe JM, Cowlishaw G 2013 How do foragers decide when to leave a patch? A test of alternative models under natural and experimental conditions. J. Anim. Ecol. 82, 894–902. (10.1111/1365-2656.12089) 23650999

[43] Amaya-Márquez M, Hill P, Abramson C, Wells H 2014 Honey bee location- and time-linked memory use in novel foraging situations: floral color dependency. Insects 5, 243–269. (10.3390/insects5010243) 26462587 PMC4592622

[44] Valone TJ 2006 Are animals capable of Bayesian updating? An empirical review. Oikos 112, 252–259. (10.1111/j.0030-1299.2006.13465.x)

[45] Sutherland WJ, Norris KJ 2002 Behavioural models of population growth rates: implications for conservation and prediction. Phil. Trans. R. Soc. Lond. B 357, 1273–1284. (10.1098/rstb.2002.1127) 12396518 PMC1693031

[46] Stillman RA, Goss-Custard JD, West AD, Durell SE, Caldow RWG, McGrorty S, Clarke RT 2000 Predicting mortality in novel environments: tests and sensitivity of a behaviour-based model. J. Appl. Ecol. 37, 564–588. (10.1046/j.1365-2664.2000.00506.x)

[47] Bolnick DI et al. 2011 Why intraspecific trait variation matters in community ecology. Trends Ecol. Evol. 26, 183–192. (10.1016/j.tree.2011.01.009) 21367482 PMC3088364

