# Supplementary material for: Information use and resource competition: an integrative framework
Source: Proc Biol Sci. 2016 Feb 24;283(1825):20152550. doi: 10.1098/rspb.2015.2550 (PMC4810826; doi:10.1098/rspb.2015.2550)
Supplement: Electronic Supplementary Material [file rspb20152550supp1.pdf]

Electronic Supplementary Material

Lee, AEG, Ounsley, JP, Coulson T, Rowcliffe, JM & Cowlishaw, G. Information use and resource competition: an integrative framework. *Proc. R. Soc. B.*

To demonstrate the generality and robustness of our key findings to changing group size, we generated numerical results for a pertinent parameter range for group sizes of 12, 20, and 28, in addition to the comprehensively explored parameter space for groups of size 4, 8, and 16 highlighted in the main manuscript. (A positive association between group size and computational demand – driven by the exponential increase in unique combinations of scroungers of different competitive weights at larger group sizes – meant that we could not generate results across a wider parameter range for group sizes larger than 16.) Here we demonstrate the robustness of our principal predictions regarding the interactive effects of resource scarcity and resource monopolisability on dominance rank-related variation in social information use. Specifically, we show that the qualitative patterns described in the main text (illustrated with data for groups of 16 individuals) also hold across groups of 4-28 individuals, with respect to the effects of resource scarcity and monopolisability on [1] the levels of scrounging (Fig. S1, cf. Fig. 1), [2] the probability of scrounging for individuals of different social rank (Fig. S2, cf. Fig. 2), [3] the skew and strength of the fitness benefits of social rank (Fig. S3, cf. Fig. 4), and [4] the group's average resource consumption rate (Fig. S4, cf. Fig. 5).

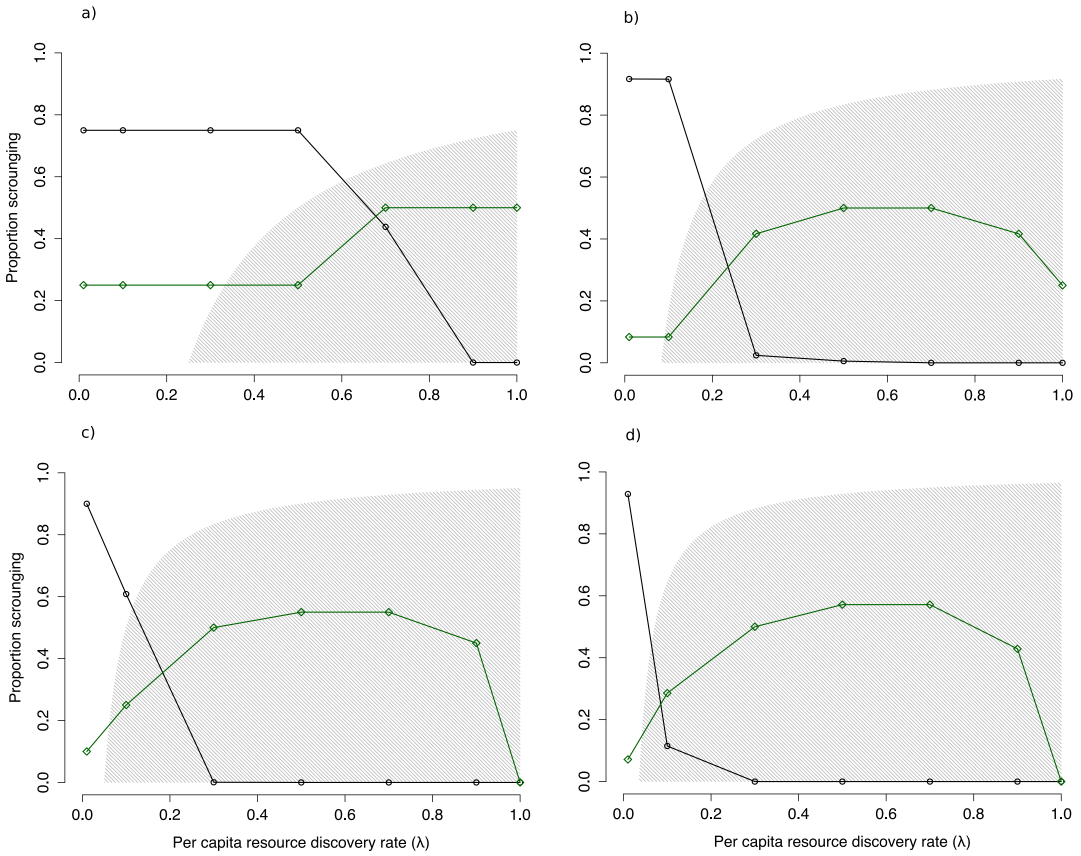

Figure S1. The combined influence of resource scarcity and monopolisability on levels of scrounging for groups of size a) 4, b) 12, c) 20, and d) 28. Simultaneous resource discoveries occurred only within the shaded region (i.e., where $\lambda q N > 1$). Values of *c* as follows: 0 (black circles); 100 (green diamonds). a/F = 0.05.

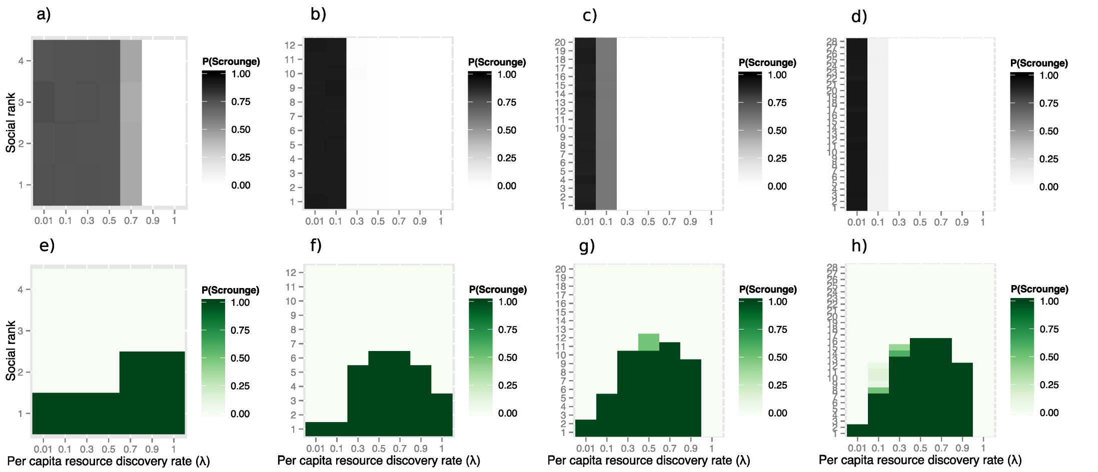

Figure S2. The combined effect of resource scarcity and monopolisability on the probability of scrounging for individuals of different social rank for groups of size a) and e) 4, b) and f) 12, c) and g) 20, and d) and h) 28. Resource monopolisability for each panel as follows: a)–d) *c* = 0 (black), e)–f) *c* = 100 (green). Colour coding corresponds to that given in Figure 1. a/F = 0.05.

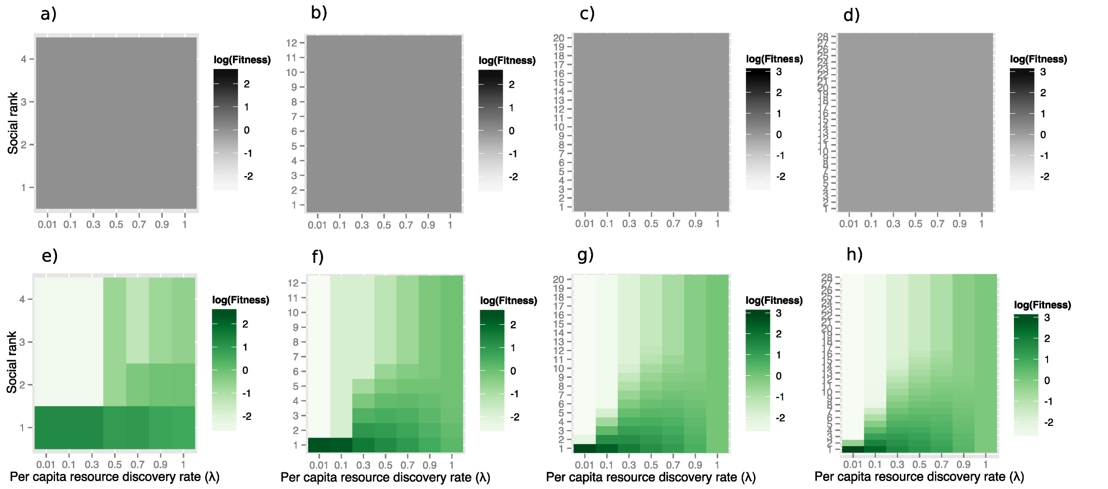

Figure S3. Resource scarcity and monopolisability interact to determine both the skew and strength of the fitness benefits of social rank, shown for groups of size a) and e) 4, b) and f) 12, c) and g) 20, and d) and h) 28. Resource monopolisability for each panel as follows: a)–d) *c* = 0 (black), e)–f) *c* = 100 (green). Colour coding corresponds to that given in Figure 1. a/F = 0.05.

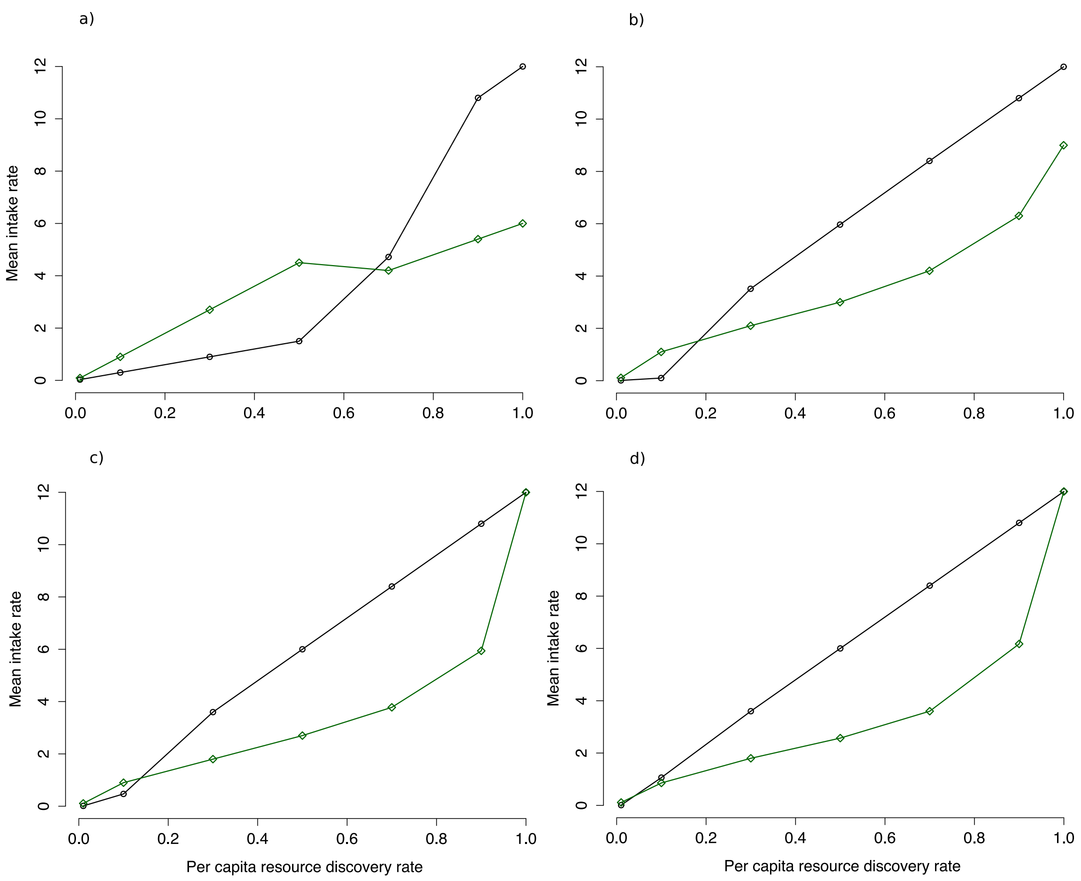

Figure S4. Resource scarcity and monopolisability affects a group's average resource consumption rate through their influence on scrounging behaviour, shown for groups of size a) 4, b) 12, c) 20, and d) 28. Values of *c* as follows: 0 (black circles); 100 (green diamonds). a/F = 0.05.
